# Exercise Addiction and Its Relationship with Health Outcomes in Indoor Cycling Practitioners in Fitness Centers

**DOI:** 10.3390/ijerph17114159

**Published:** 2020-06-11

**Authors:** Javier Bueno-Antequera, Carmen Mayolas-Pi, Joaquin Reverter-Masià, Isaac López-Laval, Miguel Ángel Oviedo-Caro, Diego Munguía-Izquierdo, Mercedes Ruidíaz-Peña, Alejandro Legaz-Arrese

**Affiliations:** 1 Physical Performance and Sports Research Center, Department of Sports and Computer Science, Section of Physical Education and Sports, Faculty of Sports Sciences, Universidad Pablo de Olavide, ES-41013 Seville, Spain; jbueant@upo.es (J.B.-A.); dmunizq@upo.es (D.M.-I.); 2 Research Group in Development Movimiento Humano, Universidad de Zaragoza, ES-50009 Zaragoza, Spain; carmayo@unizar.es (C.M.-P.); reverter@didesp.udl.cat (J.R.-M.); isaac@unizar.es (I.L.-L.); mruidiaz@unizar.es (M.R.-P.); alegaz@unizar.es (A.L.-A.); 3 Department of Physiatry and Nursery, Section of Physical Education and Sports ES-28029, Faculty of Health and Sport Sciences, University of Zaragoza, ES-50009 Zaragoza, Spain; 4 Section of Physical Education and Sports, Faculty of Education, Psychology and Social Work, University of Lleida, ES-25001 Lleida, Spain; 5 Biomedical Research Networking, Center on Frailty and Healthy Aging, ES-28029 Madrid, Spain

**Keywords:** exercise addiction, indoor cycling, health outcomes, fitness, anxiety

## Abstract

We studied the prevalence and possible association between exercise addiction and health in indoor cycling practitioners. In 1014 (492 women) adult indoor cyclists and 926 (597 women) controls with low levels of physical activity according to the short form of the International Physical Activity Questionnaire, we examined the risk of exercise addiction according to the Exercise Addiction Inventory and several health outcomes through a web-based experiment. The prevalence of a high risk of exercise addiction in cyclists was 13.3%, and it was higher in men than in women (16.5% vs. 10.0%, *p* = 0.002). Women cyclists with a high risk of exercise addiction had higher levels of physical activity (*p* < 0.001; effect size = −0.62, 95% CI: (−0.91, −0.32)) and anxiety symptom severity (*p* = 0.001; Effect Size (ES) = −0.59 (−0.89, −0.30)) than those with a low risk. For both sexes, cyclists with a low risk of exercise addiction had better social function, emotional role, and anxiety symptom severity compared with the controls (all *p* < 0.002; ES ranged from 0.25 to 0.47). Higher anxiety symptom severity and cardiorespiratory fitness were the main determinants of exercise addiction in cyclists (both *p* < 0.001). Our data suggest the importance of considering exercise addiction in indoor cyclists.

## 1. Introduction

Exercise addiction, defined as “an abnormal reliance on exercise behavior to cope with chronic stress or the hassles and challenges of the everyday life and featuring the core components of addiction found in more traditional addictions” [1], is a popular but relatively new research topic. Many researchers claim that exercise addiction should be accepted as a mental disorder in diagnostic manuals; however, the accumulated literature is still scarce and limited by a lack of conceptual and methodological consistency [1].

Two key factors to consider in improving the understanding of exercise addiction are its prevalence and health consequences. The sports discipline and the screening scale employed affect the results regarding prevalence [2,3]. Along these lines, a recent systematic review [2] suggested that endurance sports (e.g., cycling) seem to be associated with a higher risk of exercise addiction than power (e.g., weightlifting), mixed (e.g., soccer), and health and fitness disciplines among fitness center users. Additionally, one study [2] recommended the use of the Exercise Addiction Inventory because it is a brief, internationally validated [4], widely used, and highly sensitive screening instrument to detect individuals at risk of exercise addiction. Researchers should use adequate tools to assess the prevalence of exercise addiction in those who engage in specific sports disciplines.

Most studies on exercise addiction have been conducted with fitness center users (e.g., [5,6]). While helpful, these studies include heterogeneous groups of exercisers that practice different disciplines and provide heterogeneous results [2]. Therefore, a key factor for improving our understanding of the impact of exercise addiction is to develop scientific studies in homogeneous samples that are specific for a single exercise discipline. To know which exercise disciplines may be potentially associated with an increased risk of exercise addiction could help to develop better exercise practices to prevent and/or treat exercise addiction. To the best of our knowledge, the study by Lichtenstein and Jensen [7] was the only prior study that used a sample of gym users who practiced a specific fitness discipline such as CrossFit. Other of the most popular specific disciplines in fitness centers with thousands affiliated that may benefit from these kinds of research advances is indoor cycling. This study aimed to explore the prevalence of exercise addiction and its possible association with health outcomes in indoor cycling practitioners. As with other addictions, exercise addiction may co-occur with other behavioral dysfunctions related to diet, smoking, or alcohol consumption and may have a negative impact on physical and mental health outcomes, such as quality of life and sleep, anxiety, depression, fitness, and body mass index. Based on a recent meta-analysis [6], we hypothesized that a high risk of exercise addiction could be associated with worse mental health in indoor cycling practitioners.

## 2. Materials and Methods

### 2.1. Participants

This cross-sectional study was part of a research project focused on exploring health and its determinants among amateur endurance outdoor cyclists, indoor cycling practitioners, and controls [8,9,10] through a web-based experiment on a freely available webpage. This study included respondents who were 18–65 years old and who engaged in a minimum of 2 h/week and 6 continuous months of indoor cycling practice in fitness centers (indoor cycling practitioners) or who were classified as having low levels of physical activity according to the Spanish short-form version of the International Physical Activity Questionnaire [11] (controls).

### 2.2. Procedures

An invitation to participate in the study was sent via e-mail to the representatives of the 4627 fitness centers officially registered in Spain. The study was also made known through media coverage, including TV and social media such as Twitter, Facebook, and LinkedIn. The invitation included a brief introduction to the study, an explanation of the anonymous and voluntary nature of participation in the study, and the link to the experiment. The experiment included questions on health behaviors and health status and took an average of 40 min to complete. At the end of the experiment, individuals needed to provide informed consent for the scientific use of the data and provide an email address to receive the results. Data were collected in the last week of May from 2016 to 2018 with the same procedure throughout the study period. To avoid duplicate samples, cases that contained the same email address were eliminated. In 2017 and 2018 we included a warning message before starting the experiment indicating that those who had already completed it should not do it again. Data analysis was conducted in February 2019. The protocol was approved by the Committee on Biomedical Ethics of the Aragón Government (PI17/0252). There was no compensation for participation.

### 2.3. Measures

#### 2.3.1. Exercise Addiction

The risk of exercise addiction was measured with the Spanish version [12] of the Exercise Addiction Inventory [13], which has satisfactory psychometric properties in the Spanish population (Cronbach’s α = 0.70 and intraclass correlation coefficient = 0.92) [12]. The inventory, which can be found in the aforementioned free full-text articles, is composed of six items with 5-point Likert-scale responses (1 = strongly disagree, 5 = strongly agree), with scores ≥ 24 indicating a high risk of addiction.

#### 2.3.2. Health Behaviors

The indoor cycling training variables were volume (hours/week) and frequency (days/week) in the last month and experience (years). Physical activity was measured with the Spanish short-form version of the International Physical Activity Questionnaire [11] (freely available on the IPAQ website) by totaling the walking, moderate- and vigorous-intensity activities and expressing the result in metabolic equivalent of task-min/week. The Spanish short-form version of the International Physical Activity Questionnaire has been validated in the Spanish population showing a correlation coefficient of 0.51 (95% CI: 0.30–0.70) for total amount of physical activity against accelerometry measures [14]. Adherence to the Mediterranean diet was assessed with the Spanish version of the 14-Item Mediterranean Diet Tool [15], with higher scores indicating higher adherence. This Spanish version of the 14-Item Mediterranean Diet Tool has been validated in the Spanish population showing a correlation coefficient of 0.52 and an intraclass correlation coefficient of 0.51 (both *p* < 0.001) for total score against the 137-item Food Frequency Questionnaire [16]. Smoking dependence was evaluated with the Spanish version (α value = 0.66) [17] of the revised version of the Fagerström Test for Nicotine Dependence [18], with lower scores indicating lower levels of dependence. Alcohol use was calculated by transforming the volumes of beer, wine, and spirits consumed in the last week into standard alcohol units [19], with lower units indicating lower levels of alcohol consumption.

#### 2.3.3. Health Status

Quality of life was measured with the Spanish version [20] of the 12-Item Health Survey 2.0 [21], which examines eight domains constituting the physical and mental component summary scores, with higher scores indicating better quality of life. The 12-Item Health Survey showed a high criterion validity because it explained more than 90% of the variability in the physical and mental component summary scores on the 36-Item Health Survey [21]. Quality of sleep was assessed with the Spanish version [22] of the Pittsburgh Sleep Quality Index [23], which examines seven sleep components that yield a global score, with lower scores indicating better quality of sleep. The Spanish version of the Pittsburgh Sleep Quality Index has satisfactory psychometric properties in the Spanish population (Cronbach’s α values ranged from 0.67 to 0.69 and test-retest correlation coefficient for global score = 0.51; *p* < 0.001) [24]. Anxiety and depression symptom severity were separately evaluated with the Spanish version [25] of the Hospital Anxiety and Depression Scale [26], with lower scores indicating lesser symptom severity. The Spanish version of the Hospital Anxiety and Depression Scale showed satisfactory psychometric properties in the Spanish population including a good internal consistency (Cronbach’s α = 0.85 for the anxiety subscale and 0.84 for the depression subscale) [25]. Body mass index was calculated using weight and height. The Spanish version of the International Fitness Scale [27] was used to measure the physical fitness level (overall and specific components: cardiorespiratory fitness, muscular strength, speed-agility, and flexibility). The individuals were asked how they perceived their own level compared with their friends’ physical fitness using a 5-point Likert-scale (very poor = 1, poor = 2, average = 3, good = 4, and very good = 5). The Spanish version of the International Fitness Scale showed satisfactory psychometric properties in the Spanish adults including test–retest reliability (averaged weighted Kappa of 0.59) and validity against objective measures [28].

#### 2.3.4. Sociodemographic Data

Sex, age, educational status, occupational status, marital status, and number of children were determined.

### 2.4. Statistical Analysis

The characteristics of indoor cycling practitioners with low and high levels of the risk of exercise addiction and controls stratified by sex were compared with chi-square test, unpaired *t*-test, and one-way ANOVA followed by a Bonferroni post hoc test, according to the nature of the variable. Possible interactions with sex were tested using two-way ANOVA. To minimize the risk of type I statistical error, analyses were corrected for multiple comparisons with the Bonferroni method, in which the threshold *p*-Value is obtained by dividing 0.05 by the number of comparisons; in this case, 0.050 × 32^−1^ = 0.002. Pearson correlation coefficients were calculated between the risk of exercise addiction and the characteristics of all indoor cycling practitioners. Multiple stepwise regression model analysis was applied to examine the possible determinants of the risk of exercise addiction (dependent variable) and significant correlates from the univariate tests (independent variables). Residuals were tested for normality, linearity, and homoscedasticity. The variance inflation factor never exceeded three, indicating that multicollinearity was not a concern [29]. Data were analyzed using SPSS Statistics for Windows, Version 22.0 (IBM Corp, Armonk, NY, USA), with statistical significance set at α = 0.050. Additionally, the standardized mean difference was quantified with the unbiased version of Cohen’s *d* (*d*_unb_), also known as Hedges’ *g*, and associated 95% confidence intervals using an online effect size calculator (http://www.cem.org/effect-size-calculator). The *d*_unb_ was calculated through formula 4 provided by Lakens [30].

## 3. Results

Of the 8257 respondents, 1014 individuals met the inclusion criteria for indoor cycling practitioners, and 926 were controls. The prevalence of a high risk of exercise addiction in indoor cycling practitioners was 13.3% (135/1014), and it was higher in men (16.5%; 86/522) than in women (10.0%; 49/492) (*p* = 0.002). The characteristics of men and women cycling practitioners and controls are presented in Table 1 and Table 2, respectively.

Women cycling practitioners with a high risk of exercise addiction had significantly higher levels of weekly training hours (*p* = 0.001; *d*_unb_ = −0.53 95% CI: (−0.82, −0.23)) and days (*p* = 0.001; *d*_unb_ = −0.56 (−0.85, −0.26)), physical activity (*p* < 0.001; *d*_unb_ = −0.62 (−0.91, −0.32)), and anxiety symptom severity (*p* = 0.001; *d*_unb_ = −0.59 (−0.89, −0.30)) than those with a low risk of exercise addiction. Independent of sex and risk of exercise addiction, indoor cycling practitioners reported better values for most of the health outcomes than the controls; however, only indoor cycling practitioners with a low risk of exercise addiction showed significant results for certain outcomes. Specifically, in men, physical role (*p* < 0.001; *d*_unb_ = 0.36 (0.20, 0.52)), social function (*p* < 0.001; *d*_unb_ = 0.37 (0.21, 0.53)), emotional role (*p* < 0.001; *d*_unb_ = 0.30 (0.14, 0.46)), and anxiety symptom severity (*p* < 0.001; *d*_unb_ = −0.47 (−0.63, −0.31)); and in women with the outcomes adherence to the Mediterranean diet (*p* < 0.001; *d*_unb_ = 0.48 (0.36, 0.61)), smoking dependence (*p* < 0.001; *d*_unb_ = −0.31 (−0.44, −0.19)), pain (*p* < 0.001; *d*_unb_ = 0.42 (0.30, 0.54)), social function (*p* < 0.001; *d*_unb_ = 0.39 (0.26, 0.51)), emotional role (*p* < 0.001; *d*_unb_ = 0.25 (0.12, 0.37)), mental health (*p* < 0.001; *d*_unb_ = 0.36 (0.24, 0.49), mental component summary (*p* < 0.001; *d*_unb_ = 0.52 (0.40, 0.65)), quality of sleep (*p* < 0.001; *d*_unb_ = −0.24 (−0.36, −0.12)), anxiety (*p* < 0.001; *d*_unb_ = −0.44 (−0.56, −0.31)) and depression (*p* < 0.001; *d*_unb_ = −0.40 (−0.52, −0.28)) symptom severity, and body mass index (*p* < 0.001; *d*_unb_ = −0.28 (−0.41, −0.16)). The two-way ANOVA showed an interaction of sex with physical activity (*p* < 0.001) and muscular strength (*p* < 0.002).

Table 3 shows the descriptive data (mean and standard deviation or percentage) and the simple correlation models for the associations of the risk of exercise addiction and the characteristics of all indoor cycling practitioners. Table 4 presents the significant final model that emerged for indoor cycling practitioners. Sex, age, physical activity, anxiety, cardiorespiratory fitness, and muscular strength were identified as determinants, explaining 13.9% of the variance in the risk of exercise addiction.

## 4. Discussion

Two major findings of the present study can be highlighted. First, 13.3% of indoor cycling practitioners in fitness centers reported a high risk of exercise addiction. Second, male sex, lower age, higher level of physical activity, muscular strength, and, especially, higher level of anxiety symptom severity and cardiorespiratory fitness were associated with a higher risk of exercise addiction.

The prevalence of exercise addiction for the overall sample of indoor cycling practitioners is similar to the mean reported in systematic reviews of studies that used the Exercise Addiction Inventory in habitual exercisers (13.2%) [6] and in endurance athletes (14.2%) [2], including one study in amateur endurance outdoor cyclists (17%) [8], but it was slightly higher than that reported in studies in gym users (8.2%) [2], which ranged from 3.6% [31] to 42.5% [32]. Although the aforementioned studies in gym users might be more useful for contextualizing our results, all included heterogeneous samples that were not specific to a particular discipline. The study of Lichtenstein and Jensen [7] showed that the prevalence of exercise addiction in people engaging in a specific fitness discipline such as CrossFit according to the Exercise Addiction Inventory was 5%, suggesting that the problem is less prevalent than among those who engage in indoor cycling. More studies in homogeneous samples that are specific for a single fitness discipline are needed to understand which disciplines are potentially associated with an increased risk of exercise addiction.

The higher prevalence of a high risk of exercise addiction in men than in women is consistent with the findings of two studies in gym users [5,32] but seems to contradict the study by Lichtenstein et al. [33] that showed no sex differences in gym users. According to studies of regular exercisers in sports clubs and leisure centers [34] and long-term fitness club members [35], we suggest that men could be more motivated than women by mastery and competition, which seem to be associated with exercise addiction [36]. Nevertheless, research on whether motivation to exercise actually explains sex differences in the prevalence rate of exercise addiction in indoor cycling practitioners is needed.

Our finding that women indoor cycling practitioners with a high risk of exercise addiction had more severe anxiety symptoms than those with a low risk is consistent with previous studies in college students who habitually engaged in exercise [37], outdoor cyclists [8], and gym users [5]. However, the finding of higher levels of fitness in the group with a high risk of exercise addiction than in the group with a low risk of exercise addiction is inconsistent with the finding of the abovementioned study in outdoor cyclists [8], which was the only study prior to ours that explored the possible association between exercise addiction and fitness. Again, possible differences in attitudes towards exercise among participants in the current study may explain these discrepancies. More research that compares the possible association of exercise addiction and fitness between “more” and “less” competitive indoor cycling practitioners is needed. Of interest, e-racing, which is a recent development in indoor cycling that involves social media, could be a possible future enabler of exercise addiction due to the competitive nature and use of interactive rewards, unlike traditional methods of indoor cycling.

The positive association between exercise addiction and age in indoor cycling practitioners is consistent with the findings of studies in habitual exercisers [6] and people who practice CrossFit [7]. Finally, our finding that cardiorespiratory fitness was, along with anxiety, the main determinant of exercise addiction in indoor cycling practitioners suggests the importance of considering fitness as a means of identifying indoor cycling practitioners who are potentially addicted to exercise. This result could be partially due to the higher volume and frequency of training and level of physical activity observed in the group of women with a high risk of exercise addiction compared with those with a low risk of exercise addiction. However, the use of a self-report-based cardiorespiratory fitness measurement limits the accuracy of our findings and suggests caution with this claim. Additionally, is important to consider possible inconsistent interpretations of the Exercise Addiction Inventory related to the nature of the studied sample as explained by Szabo et al. [36]. It may be possible that some of our sample of indoor cyclists may have confounded exercise addiction (associated with adverse effects on health) [6] with exercise harmonious passion (associated with beneficial effects on health) [38].

### 4.1. Interpretation and Practical Application of Current Research Findings

The current study could help raise awareness among health professionals of the importance of considering exercise addiction as a health risk factor in indoor cycling practitioners and may encourage the scientific community to research the prevalence and effects of exercise addiction in homogeneous samples that are specific for a single exercise discipline. This study identified determinants of exercise addiction that should be considered. However, our model explained only approximately 14% of the variance in the risk of exercise addiction, suggesting that other outcomes not included in this study, such as attitudes towards exercise, passion [38], narcissism, self-esteem [32], eating disorders [6,39], emotional distress [40], body appearance anxiety, and use of fitness supplements [5], could explain some of the remaining variance. Better indoor cycling practices may be needed to prevent and/or treat exercise addiction. Multidisciplinary teams including qualified exercise professionals should play an important role in addressing exercise addiction.

### 4.2. Limitations and Strengths

The two major limitations were that the study was cross-sectional in design and based on self-reported data, which preclude causal inferences [41] and involve bias [42], respectively. Other aspects that may also limit generalization [43] were the following: (1) we do not know whether the representatives of fitness centers sent the information to all users, the number of people who declined to participate, the reasons for refusal to participate, or the intensity of the indoor cycling training sessions; (2) indoor cycling practitioners may undertake other physical activities; (3) among the small number of participants with a high risk of exercise addiction, few were women.

Despite these limitations, this study has strengths. The Internet design of the experiment, media coverage, and anonymous participation prevented problems of missing data [44], increased access to more potential participants, and may have encouraged individuals to respond to very personal questions [45]. Furthermore, well-established, validated, and reliable measures that apply norm-based scoring methodology were used (for details, see Section 2).

## 5. Highlights

- A total of 13.3% of indoor cycling practitioners reported a high risk of exercise addiction.
- Exercise addiction was more prevalent in men and associated with worse health outcomes.
- Cardiorespiratory fitness and anxiety were the main determinants of exercise addiction.

## 6. Conclusions

In total, 13.3% of indoor cycling practitioners exhibited a high risk of exercise addiction; the proportion was higher in men and was associated with more severe anxiety symptoms in women. Independent of sex and the risk of exercise addiction, indoor cycling practitioners reported better values for most of the health outcomes than the controls. However, only indoor cycling practitioners with a low risk of exercise addiction showed significant results for certain outcomes, such as social function, emotional role, and anxiety symptom severity. In addition, anxiety and cardiorespiratory fitness were identified as major determinants of exercise addiction. Finally, the findings on anxiety seems to confirm our principal hypothesis that a high risk of exercise addiction could be associated with worse mental health in indoor cycling practitioners.

## Figures and Tables

**Table 1:** Characteristics of men cycling indoor practitioners and controls.

Variables	Indoor Cycling (*n* = 522)		Control (*n* = 329)	Low vs. High REA		High REA vs. Control		Low REA vs. Control		ANOVA
	Low REA (*n* = 436)	High REA (*n* = 86)		*p*-Value	*d*_unb_ (95% CI)	*p*-Value	*d*_unb_ (95% CI)	*p*-Value	*d*_unb_ (95% CI)	*p*-Value
Educational status (non-university studies)	229 (52.5)	45 (52.3)	190 (57.8)	1.000		0.394		0.164		
Occupational status (unemployed)	68 (15.6)	15 (17.4)	92 (28)	0.633		0.053		**<0.001**		
Marital status (single)	102 (23.4)	15 (17.7)	100 (30.4)	0.259		0.021		0.032		
Number of children (≥1)	216 (49.5)	42 (48.8)	150 (45.6)	0.907		0.628		0.306		
Training volume (h/week)	3.1 ± 1.2	3.1 ± 1.1		0.855	−0.02 (−0.25, 0.21)					
Training frequency (d/week)	3.0 ± 1.1	3.0 ± 1.0		0.833	0.02 (−0.21, 0.26)					
Experience in indoor cycling (years)	4.1 ± 3.7	4.4 ± 4.0		0.492	−0.08 (-0.31, 0.15)					
Age (years)	39.9 ± 10.7	38.1 ± 9.5	37.2 ± 12.8	0.527	0.17 (−0.06, 0.41)	1.000	0.07 (−0.17, 0.32)	0.004	0.24 (0.08, 0.40)	0.005
Exercise addiction (6–30)	17.6 ± 3.8	25.7 ± 1.7	11.7 ± 5.1	**<0.001**	−2.29 (−2.56, −2.02)	**<0.001**	3.13 (2.79, 3.48)	**<0.001**	1.37 (1.19, 1.54)	**<0.001**
Physical activity (MET-min/week)	4517.6 ± 2930.2	4986.0 ± 2857.6	502.7 ± 674.7	0.264	−0.16 (−0.39, 0.07)	**<0.001**	2.83 (2.50, 3.16)	**<0.001**	1.68 (1.50, 1.86)	**<0.001**
Adherence to the Mediterranean diet (0–14)	8.2 ± 2.1	8.3 ± 1.8	7.1 ± 2.2	1.000	−0.04 (−0.27, 0.19)	**<0.001**	0.53 (0.28, 0.78)	**<0.001**	0.49 (0.33, 0.65)	**<0.001**
Smoking dependence (0–6)	0.48 ± 1.63	0.33 ± 1.47	1.39 ± 2.76	1.000	0.09 (−0.14, 0.33)	**<0.001**	−0.43 (−0.68, −0.18)	**<0.001**	−0.43 (−0.59, −0.27)	**<0.001**
Alcohol use (SAU/weeek)	6.8 ± 8.3	4.8 ± 6.5	7.7 ± 10.0	0.148	0.26 (0.02, 0.49)	0.020	−0.31 (−0.56, −0.07)	0.555	−0.10 (−0.25, 0.06)	0.023
Quality of life (0–100)										
Physical function	55.7 ± 3.7	55.2 ± 4.8	50.4 ± 9.2	1.000	0.14 (−0.10, 0.37)	**<0.001**	0.57 (0.32, 0.82)	**<0.001**	0.84 (0.68, 1.01)	**<0.001**
Physical role	54.3 ± 8.4	52.3 ± 10.4	50.8 ± 11.4	0.253	0.23 (0.00, 0.46)	0.724	0.13 (−0.12, 0.37)	**<0.001**	0.36 (0.20, 0.52)	**<0.001**
Pain	54.8 ± 5.9	54.7 ± 6.3	51.6 ± 8.8	1.000	0.02 (−0.21, 0.25)	0.001	0.37 (0.12, 0.62)	**<0.001**	0.45 (0.29, 0.61)	**<0.001**
General health	51.6 ± 7.0	53.3 ± 7.2	44.9 ± 9.4	0.248	−0.23 (−0.47, 0.00)	**<0.001**	0.94 (0.68, 1.20)	**<0.001**	0.85 (0.68, 1.01)	**<0.001**
Vitality	56.7 ± 8.2	58.9 ± 8.4	52.8 ± 9.8	0.115	−0.26 (−0.49, −0.03)	**<0.001**	0.65 (0.40, 0.90)	**<0.001**	0.45 (0.29, 0.61)	**<0.001**
Social function	51.7 ± 8.2	50.2 ± 10.0	48.4 ± 10.2	0.551	0.17 (−0.06, 0.40)	0.277	0.18 (−0.06, 0.43)	**<0.001**	0.37 (0.21, 0.53)	**<0.001**
Emotional role	49.7 ± 14.0	48.8 ± 15.1	45.2 ± 17.1	1.000	0.06 (−0.17, 0.30)	0.162	0.22 (−0.03, 0.46)	**<0.001**	0.30 (0.14, 0.46)	**<0.001**
Mental health	52.1 ± 8.4	50.4 ± 8.6	50.4 ± 8.6	0.246	0.21 (−0.02, 0.44)	1.000	0.00 (−0.25, 0.25)	0.015	0.21 (0.05, 0.36)	0.012
Physical component summary	55.8 ± 5.8	56.0 ± 7.6	50.7 ± 9.7	1.000	−0.03 (−0.26, 0.20)	**<0.001**	0.58 (0.33, 0.83)	**<0.001**	0.69 (0.53, 0.85)	**<0.001**
Mental component summary	51.1 ± 13.7	51.4 ± 14.1	42.0 ± 17.0	1.000	−0.02 (−0.25, 0.21)	**<0.001**	0.57 (0.32, 0.82)	**<0.001**	0.61 (0.45, 0.77)	**<0.001**
Quality of sleep (0–21)	4.6 ± 2.5	4.6 ± 2.6	5.3 ± 2.9	1.000	−0.02 (−0.25, 0.21)	0.158	−0.22 (−0.47, 0.03)	0.001	−0.26 (−0.42, −0.10)	0.002
Anxiety (0–21)	8.5 ± 2.4	9.3 ± 3.0	9.7 ± 3.0	0.039	−0.32 (−0.55, −0.08)	0.503	−0.15 (−0.40, 0.10)	**<0.001**	−0.47 (−0.63, −0.31)	**<0.001**
Depression (0–21)	9.5 ± 2.2	9.4 ± 2.3	10.2 ± 2.9	1.000	0.05 (−0.18, 0.28)	0.052	−0.26 (−0.51, −0.02)	0.003	−0.25 (−0.40, −0.09)	0.002
Body mass index (kg/m^2^)	25.5 ± 2.8	25.1 ± 3.3	25.8 ± 3.8	0.980	0.13 (−0.10, 0.36)	0.205	−0.20 (−0.44, 0.05)	0.451	−0.11 (−0.27, 0.05)	0.131
Fitness (1–5)										
Overall	3.9 ± 0.7	4.2 ± 0.7	2.9 ± 0.8	0.012	−0.36 (−0.59, −0.13)	**<0.001**	1.54 (1.26, 1.81)	**<0.001**	1.28 (1.10, 1.45)	**<0.001**
Cardiorespiratory fitness	3.8 ± 0.8	4.1 ± 0.8	2.8 ± 0.9	0.019	−0.35 (−0.58, −0.11)	**<0.001**	1.43 (1.16, 1.70)	**<0.001**	1.23 (1.06, 1.40)	**<0.001**
Muscular strength	3.7 ± 0.7	3.9 ± 0.8	3.2 ± 0.8	0.065	−0.29 (−0.52, −0.05)	**<0.001**	0.92 (0.66, 1.17)	**<0.001**	0.70 (0.54, 0.86)	**<0.001**
Speed-agility	3.5 ± 0.8	3.7 ± 0.8	2.8 ± 0.9	0.074	−0.28 (−0.52, −0.05)	**<0.001**	1.04 (0.78, 1.30)	**<0.001**	0.85 (0.69, 1.02)	**<0.001**
Flexibility	3.0 ± 0.8	3.1 ± 1.0	2.4 ± 1.0	0.727	−0.15 (−0.38, 0.09)	**<0.001**	0.69 (0.44, 0.94)	**<0.001**	0.63 (0.47, 0.79)	**<0.001**

Values are the mean ± standard deviation or *n* (%); CI, confidence intervals; *d*_unb_, unbiased version of Cohen’s *d*; MET, metabolic equivalent of task; REA, risk of exercise addiction; SAU, standard alcohol units’; chi-square test was used to examine differences in the proportion of comparing educational, occupational, and marital status and number of children; unpaired *t*-test was used to examine differences of training volume and frequency and experience in indoor cycling; one-way ANOVA with Bonferroni post hoc test was used to examine differences of resting variables; significant *p*-Values are in bold; the threshold *p*-Value was set at 0.002 (=0.050/32).

**Table 2:** Characteristics of women cycling indoor practitioners and controls.

Variables	Indoor Cycling (*n* = 492)		Control (*n* = 597)	Low vs. High REA		High REA vs. Control		Low REA vs. Control		ANOVA
	Low REA (*n* = 443)	High REA (*n* = 49)		*p*-Value	*d*_unb_ (95%CI)	*p*-Value	*d*_unb_ (95% CI)	*p*-Value	*d*_unb_ (95% CI)	*p*-Value
Educational status (non-university studies)	133 (30)	17 (34.7)	240 (40.2)	0.515		0.544		0.001		
Occupational status (unemployed)	138 (31.2)	22 (44.9)	262 (43.9)	0.055		0.882		**<0.001**		
Marital status (single)	85 (19.2)	21 (42.9)	146 (24.5)	**<0.001**		0.007		0.050		
Number of children (≥1)	173 (39.1)	15 (30.6)	253 (42.4)	0.281		0.131		0.308		
Training volume (h/week)	2.7 ± 0.9	3.2 ± 1.3			−0.53 (−0.82, −0.23)					0.001
Training frequency (d/week)	2.7 ± 0.9	3.2 ± 1.3			−0.56 (−0.85, −0.26)					**<0.001**
Experience in indoor cycling (years)	3.5 ± 3.5	4.3 ± 5.3			−0.22 (−0.52, 0.07)					0.135
Age (years)	35.2 ± 10.9	32.2 ± 8.9	34.5 ± 12.7	0.301	0.27 (−0.02, 0.57)	0.615	−0.18 (−0.47, 0.11)	1.000	0.06 (−0.06, 0.18)	0.221
Exercise addiction (6-30)	16.9 ± 3.7	25.4 ± 1.5	11.0 ± 4.6	**<0.001**	−2.39 (−2.72, −2.06)	**<0.001**	3.23 (2.89, 3.57)	**<0.001**	1.37 (1.24, 1.51)	**<0.001**
Physical activity (MET-min/week)	3564.1 ± 2406.1	5120.7 ± 3371.8	379.6 ± 367.1	**<0.001**	−0.62 (−0.91, −0.32)	**<0.001**	4.80 (4.41, 5.19)	**<0.001**	2.00 (1.85, 2.15)	**<0.001**
Adherence to the Mediterranean diet (0–14)	8.5 ± 1.8	8.3 ± 2.0	7.6 ± 2.1	1.000	0.10 (−0.19, 0.40)	0.029	0.37 (0.08, 0.66)	**<0.001**	0.48 (0.36, 0.61)	**<0.001**
Smoking dependence (0−16)	0.30 ± 1.22	0.35 ± 1.36	0.85 ± 2.06	1.000	−0.04 (−0.33, 0.26)	0.155	−0.25 (−0.54, 0.04)	**<0.001**	−0.31 (−0.44, −0.19)	**<0.001**
Alcohol use (SAU/week)	3.8 ± 7.3	2.7 ± 4.9	4.4 ± 8.3	1.000	0.16 (−0.14, 0.45)	0.387	−0.22 (−0.51, 0.07)	0.560	−0.08 (−0.20, 0.04)	0.179
Quality of life (0–100)										
Physical function	55.4 ± 4.3	55.6 ± 3.2	50.1 ± 9.0	1.000	−0.06 (−0.35, 0.24)	**<0.001**	0.63 (0.34, 0.92)	**<0.001**	0.71 (0.58, 0.84)	**<0.001**
Physical role	51.7 ± 11.2	52.3 ± 9.8	49.8 ± 12.7	1.000	−0.05 (−0.35, 0.24)	0.503	0.20 (−0.10, 0.49)	0.035	0.16 (0.03, 0.28)	0.027
Pain	54.5 ± 6.2	52.2 ± 10.6	51.1 ± 9.2	0.204	0.33 (0.04, 0.63)	1.000	0.12 (−0.17, 0.41)	**<0.001**	0.42 (0.30, 0.54)	**<0.001**
General health	51.8 ± 6.9	52.5 ± 8.3	46.1 ± 9.2	1.000	−0.10 (−0.40, 0.19)	**<0.001**	0.70 (0.41, 0.99)	**<0.001**	0.68 (0.56, 0.81)	**<0.001**
Vitality	55.0 ± 8.5	57.0 ± 9.2	51.1 ± 9.9	0.458	−0.23 (−0.53, 0.06)	**<0.001**	0.60 (0.31, 0.90)	**<0.001**	0.42 (0.30, 0.55)	**<0.001**
Social function	50.6 ± 8.6	48.5 ± 10.9	47.0 ± 10.2	0.437	0.24 (−0.06, 0.53)	0.803	0.15 (−0.14, 0.45)	**<0.001**	0.39 (0.26, 0.51)	**<0.001**
Emotional role	46.6 ± 16.4	43.3 ± 18.8	42.3 ± 18.3	0.621	0.20 (−0.09, 0.50)	1.000	0.05 (−0.24, 0.35)	**<0.001**	0.25 (0.12, 0.37)	**<0.001**
Mental health	50.5 ± 8.2	46.8 ± 10.3	47.2 ± 9.4	0.022	0.43 (0.13, 0.73)	1.000	−0.04 (−0.33, 0.25)	**<0.001**	0.36 (0.24, 0.49)	**<0.001**
Physical component summary	55.7 ± 7.0	57.2 ± 7.3	51.7 ± 10.6	0.874	−0.21 (−0.50, 0.09)	**<0.001**	0.53 (0.24, 0.82)	**<0.001**	0.44 (0.31, 0.56)	**<0.001**
Mental component summary	48.2 ± 15.4	45.3 ± 18.4	39.4 ± 17.9	0.732	0.19 (−0.11, 0.48)	0.063	0.32 (0.03, 0.62)	**<0.001**	0.52 (0.40, 0.65)	**<0.001**
Quality of sleep (0–21)	4.6 ± 2.6	5.4 ± 3.5	5.3 ± 3.2	0.301	−0.27 (−0.57, 0.02)	1.000	0.01 (−0.28, 0.30)	**<0.001**	−0.24 (−0.36, −0.12)	0.001
Anxiety (0–21)	9.0 ± 2.4	10.5 ± 3.6	10.2 ± 3.1	0.001	−0.59 (−0.89, −0.30)	1.000	0.09 (−0.20, 0.38)	**<0.001**	−0.44 (−0.56, −0.31)	**<0.001**
Depression (0–21)	9.4 ± 2.2	10.0 ± 2.5	10.4 ± 2.9	0.308	−0.29 (−0.58, 0.01)	0.884	−0.14 (−0.43, 0.15)	**<0.001**	−0.40 (−0.52, −0.28)	**<0.001**
Body mass index (kg/m^2^)	22.4 ± 3.0	22.5 ± 3.2	23.5 ± 4.3	1.000	−0.01 (−0.31, 0.28)	0.201	−0.24 (−0.53, 0.05)	**<0.001**	−0.28 (−0.41, −0.16)	**<0.001**
Fitness (1–5)										
Overall	3.8 ± 0.7	4.1 ± 0.7	2.9 ± 0.8	0.039	−0.41 (−0.70, −0.11)	**<0.001**	1.54 (1.24, 1.84)	**<0.001**	1.26 (1.13, 1.39)	**<0.001**
Cardiorespiratory fitness	3.5 ± 0.8	3.8 ± 0.8	2.4 ± 0.9	0.021	−0.41 (−0.71, −0.12)	**<0.001**	1.62 (1.31, 1.92)	**<0.001**	1.23 (1.10, 1.37)	**<0.001**
Muscular strength	3.4 ± 0.8	3.8 ± 0.9	2.6 ± 0.9	0.036	−0.40 (−0.69, −0.10)	**<0.001**	1.28 (0.98, 1.58)	**<0.001**	0.96 (0.83, 1.09)	**<0.001**
Speed–agility	3.4 ± 0.8	3.5 ± 0.7	2.6 ± 0.9	1.000	−0.14 (−0.43, 0.16)	**<0.001**	1.01 (0.71, 1.30)	**<0.001**	0.93 (0.80, 1.06)	**<0.001**
Flexibility	3.4 ± 1.0	3.6 ± 1.1	2.9 ± 1.0	0.348	−0.24 (−0.54, 0.05)	**<0.001**	0.75 (0.45, 1.04)	**<0.001**	0.53 (0.41, 0.66)	**<0.001**

Values are the mean ± standard deviation or n (%); CI, confidence intervals; *d*_unb_, unbiased version of Cohen’s *d*; MET, metabolic equivalent of task; REA, risk of exercise addiction; SAU, standard alcohol units; chi-square test was used to examine differences in the proportion of comparing educational, occupational, and marital status and number of children. Unpaired *t*-test was used to examine differences of training volume and frequency and experience in indoor cycling. One-way ANOVA with Bonferroni post hoc test was used to examine differences of resting variables; significant *p*-Values are in bold; the threshold *p*-Value was set at 0.002 (=0.050/32).

**Table 3:** Descriptive data (mean and standard deviation or percentage) and associations with risk of exercise addiction in all indoor cycling practitioners (*n* = 1014).

Variables	Descriptive	Correlation with the Risk of Exercise Addiction	*p*-Value
Sex (men/women)	492 (48.5)	−0.01	**<0.001**
Educational status (non-university/university studies)	424 (41.8)	−0.02	0.542
Occupational status (un-/employed)	243 (24.0)	−0.05	0.147
Marital status (single/couple)	223 (22.0)	0.08	**0.008**
Number of children (≥1/0)	446 (44.0)	−0.02	0.509
Training volume (h/week)	2.9 ± 1.1	0.08	**0.009**
Training frequency (d/week)	2.9 ± 1.1	0.10	**0.003**
Experience in indoor cycling (years)	3.9 ± 3.7	0.05	0.087
Exercise addiction (6–30)	18.3 ± 4.6	1.00	-
Age (years)	37.3 ± 10.9	−0.09	**0.006**
Physical activity (MET-min/week)	4169.9 ± 2783.9	0.15	**<0.001**
Adherence to the Mediterranean diet (0–14)	8.3 ± 1.9	−0.02	0.641
Smoking dependence (0–16)	0.4 ± 1.4	−0.06	0.052
Alcohol use (SAU/week)	5.1 ± 7.7	−0.02	0.565
Quality of life (0–100)			
Physical function	55.5 ± 4.0	0.03	0.402
Physical role	52.9 ± 10.0	−0.03	0.307
Pain	54.6 ± 6.4	−0.05	0.115
General health	51.9 ± 7.0	0.09	**0.006**
Vitality	56.2 ± 8.5	0.06	0.074
Social function	51.0 ± 8.7	−0.06	0.052
Emotional role	48.0 ± 15.5	−0.05	0.132
Mental health	51.0 ± 8.5	−0.07	**0.025**
Physical component summary	55.9 ± 6.6	0.05	0.116
Mental component summary	49.6 ± 14.8	−0.01	0.838
Quality of sleep (0–21)	4.6 ± 2.6	0.05	0.138
Anxiety (0–21)	8.9 ± 2.6	0.16	**<0.001**
Depression (0–21)	9.5 ± 2.2	0.04	0.262
Body mass index (kg/m^2^)	24.0 ± 3.3	−0.02	0.599
Fitness (1–5)			
Overall	3.9 ± 0.7	0.22	**<0.001**
Cardiorespiratory fitness	3.7 ± 0.8	0.27	**<0.001**
Muscular strength	3.6 ± 0.8	0.21	**<0.001**
Speed-agility	3.5 ± 0.8	0.16	**<0.001**
Flexibility	3.2 ± 0.9	0.04	0.180

Descriptive data are the mean ± standard deviation or *n* (%); MET, metabolic equivalent of task; SAU, standard alcohol units; significant when *p*-Value < 0.050 (significant *p*-Values are in bold).

**Table 4:** Final model of the backward stepwise regression analysis with the exercise addiction score as the dependent variable in cycling indoor practitioners.

Variables ^a^	B	SE	β	*t*	VIF	*p*-Value
(Constant)	11.07	1.25		8.89		<0.001
Cardiorespiratory fitness	1.13	0.19	0.21	5.97	1.46	<0.001
Anxiety	0.37	0.05	0.21	7.05	1.03	<0.001
Muscular strength	0.50	0.20	0.09	2.46	1.42	0.014
Sex (men/women)	−0.85	0.28	−0.09	−3.00	1.13	0.003
Age	−0.03	0.01	−0.08	−2.70	1.07	0.007
Physical activity	0.00	0.00	0.08	2.51	1.10	0.012

B, unstandardized coefficient; β, standardized coefficient; SE, standard error; VIF, variance inflation factor; ^a^ Only significant correlates in the Pearson correlation test were included in the model (see Table 3); significant when *p*-Value < 0.050.
